# The anti-aging effects of LW-AFC *via* correcting immune dysfunctions in senescence accelerated mouse resistant 1 (SAMR1) strain

**DOI:** 10.18632/oncotarget.8877

**Published:** 2016-04-20

**Authors:** Jianhui Wang, Xiaorui Cheng, Xiaorui Zhang, Junping Cheng, Yiran Xu, Ju Zeng, Wenxia Zhou, Yongxiang Zhang

**Affiliations:** ^1^ Department of Neuroimmunopharmacology, Beijing Institute of Pharmacology and Toxicology, Beijing, China; ^2^ State Key Laboratory of Toxicology and Medical Countermeasures, Beijing, China

**Keywords:** LW-AFC, senescence accelerated mouse resistant 1 (SAMR1) strain, immunosenescence, immunodeficiency, chronic inflammation, Gerotarget

## Abstract

Although there were considerable advances in the anti-aging medical field, it is short of therapeutic drug for anti-aging. Mounting evidence indicates that the immunosenescence is the key physiopathological mechanism of aging. This study showed the treatment of LW-AFC, an herbal medicine, decreased the grading score of senescence, increased weight, prolonged average life span and ameliorated spatial memory impairment in 12- and 24-month-old senescence accelerated mouse resistant 1 (SAMR1) strain. And these anti-aging effects of LW-AFC were more excellent than melatonin. The administration of LW-AFC enhanced ConA- and LPS-induced splenocyte proliferation in aged SAMR1 mice. The treatment of LW-AFC not only reversed the decreased the proportions of helper T cells, suppressor T cells and B cells, the increased regulatory T cells in the peripheral blood of old SAMR1 mice, but also could modulate the abnormal secretion of IL-1β, IL-2, IL-6, IL-17, IL-23, GM-CSF, IFN-γ, TNF-α, TNF-β, RANTES, eotaxin, MCP-1, IL-4, IL-5, IL-10 and G-CSF. These data indicated that LW-AFC reversed the immunosenescence status by restoring immunodeficiency and decreasing chronic inflammation and suggested LW-AFC may be an effective anti-aging agent.

## INTRODUCTION

Aging is characterized by time-dependent loss of physiological integrity, leading to impaired function and increased vulnerability to disease [1]. And aging is a complex process that deeply affected by genomic instability [2], telomere attrition [3, 4], epigenetic alterations [5, 6], loss of proteostasis [7], deregulated nutrient sensing [8, 9], mitochondrial dysfunction [10–12], cellular senescence [13], stem cell exhaustion [14, 15], etc. Because of the intrinsic complexities to the multifactorial causes underlying aging, few proposed aging interventions have received good press. Such as caloric restriction [9, 16], spermidine [17], metformin [18, 19], resveratrol [20, 21], and rapamycin [22]. However, these interventions showed significantly negative side effects, including danger of malnutrition [23], nausea, gastrointestinal discomfort, nephrotoxicity [24], potent immunosuppression [25], and adverse effects [26].

For the mechanism underlying aging, mounting evidence indicates the immune system undergoes serious deterioration with age and the immunosenescence plays the key role in aging [27, 28]. Immunosenescence has been defined as the age-associated decrease in immune competence that renders individuals more susceptible to diseases and increases morbidity and mortality due to infections and a variety of other age-related pathologies [29]. If we want to couple a longer lifespan with healthy ageing, it is imperative to develop intervention strategies to reduce the effects of immunosenescence. However, numerous kinds of cells and factors were involved in the process of immunosenescence which the key points contain progressively adaptive immune system inability and chronic inflammation with ageing [30]. Thus, when evaluating therapeutic interventions for extenuating or antagonizing transformations with age, any correction of immune system dysfunctions must be accompanied by an anti-inflammatory responses [31, 32].

LW-AFC is consisted of 20.3% polysaccharide fraction (LWB-B), 15.1% glycosides fraction (LWD-b) and 64.6% oligosaccharide fraction (CA-30), which are extracted main active components from Liuwei Dihuang decoction. Liuwei Dihuang decoction is comprised of *Rehmannia glutinosa* Libosch., *Cornus officinalis* Sieb., *Dioscorea opposita* Thunb., *Alisma orientale* (G. Samuelsson) Juz, *Poria cocos* (Schw.) Wolf and *Paeonia suffruticosa* Andrews in the weight ratio 8:4:4:3:3:3. Liuwei Dihuang decoction is a classical traditional Chinese medicine (TCM) prescription with a history of thousand years for improving or restoring declined functions related to aging process and geriatric diseases including hypertension [13], diabetes [14], osteoporosis [15], dementia [16, 17].

Senescence accelerated mouse resistant 1 (SAMR1) strain, one of three litters of mice resistant to accelerated aging was derived from AKR/J strain and established by Toshio Takeda and colleagues [10], represent a normal aging control. The senescence process of SAMR1 mice show normal development and maturation. The degree of senescence at 8-months-old SAMR1 mice is 3.4 [9], the median survival time is 568 days, these value corresponds to those of common strains [11]. In autopsy findings, SAMR1 mice show non-thymic lymphoma, histiocytic sarcoma and ovarian cysts [12]. Thus, SAMR1 mice provide an excellent experimental model control for verifying the effect of accelerated aging repeatedly.

In present study we found long-term oral administration of LW-AFC, an herbal medicine, delayed appearance of aging in old SAMR1 mice, which were more excellent than melatonin, an indoleamine as a pharmacological anti-aging intervention [42–44] with immunomodulatory activity [45, 46] and anti-inflammatory capability [47–49]. Furthermore, we found LW-AFC not only effected on immune system dysfunctions but also on inflammatory responses.

## RESULTS

### The treatment of LW-AFC slowed the aging process of old SAMR1 mice

**Figure 1:**
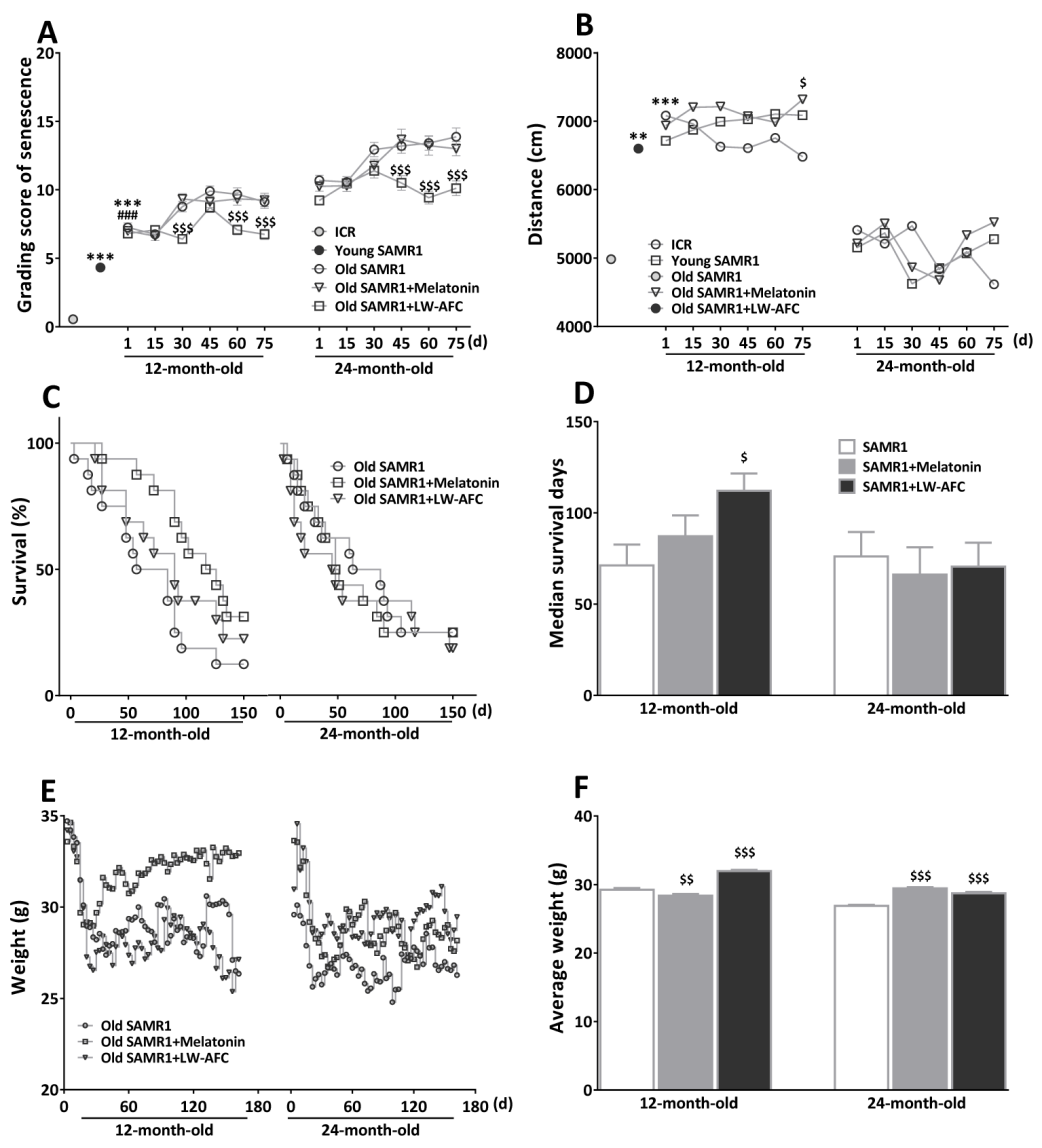
The treatment of LW-AFC slowed the aging process of SAMR1 mice **A.** The chronological change of total grading score for evaluation of aging of SAMR1 mice. The difference between control group and LW-AFC group of 12-month-old SAMR1 mice became obvious from 30 days after treatment. Regarding with 24-month-old SAMR1 mice, the difference between control group and LW-AFC group became obvious from 45 days after treatment. **B.** Effect of LW-AFC on the spontaneous locomotor activity in SAMR1 mice. The total distance of the 12-month-old and 24-month-old SAMR1 mice in spontaneous locomotor activity test, showed increased distance of 12-month-old SAMR1 mice when treated with Melatonin. **C.** Survival trajectories of 12-and 24-month-old SAMR1 mice. **D.** Median survival time of 12- and 24-month-old SAMR1 mice showed significantly increased survival of 12-month-old SAMR1 mice when treated with LW-AFC. **E.** The weight trends of SAMR1 mice. **F.** Average weight (sum of weight before decapitation/(51×sample size)) of SAMR1 mice showed significantly increased when treated with LW-AFC, decreased weight of 12-month-old SAMR1 mice and increased that of 24-month-old SAMR1 mice treated with melatonin, respectively. The values are mean ± SEM, or only mean. *n* = 2∼16. **P < 0.01, ***P < 0.001, *versus* ICR mice; ^###^*P* < 0.001, *versus* young SAMR1 mice; ^$^*P* < 0.05, ^$$^*P* < 0.01, ^$$$^*P* < 0.001, *versus* the old SAMR1 mice by unpaired Student's *t*-test and one-way ANOVA analysis followed by Dunnett's post hoc test.

In order to investigate the effects of LW-AFC on aging, we detected the influence of LW-AFC on the appearance of aging. Results showed the treatment of LW-AFC had effects on the grading score of senescence, life span and weight except for spontaneous locomotor activity (Figure 1B).

### LW-AFC had delaying effects on aging process of old SAMR1 mice

Results showed the grading score being used to evaluate the degree of senescence in 12- and 24-month-old SAMR1 mice was significantly higher than that in 7-month-old SAMR1 and ICR mice (Figure 1A). After treatment of LW-AFC, the grading score in two different month SAMR1 mice was decreased. The decreased grading score induced by the treatment of LW-AFC in 12- and 24-month-old SAMR1 mice was observed at 30 and 45 days after treatment respectively, while the treatment of melatonin could not change the grading score in 12- and 24-month-old SAMR1 mice (Figure 1A). This indicated that the treatment of LW-AFC delayed the aging process of SAMR1 mice.

### The impact of LW-AFC on the average life span and weight of old SAMR1 mice

The life span of each 12- and 24-month-old SAMR1 mouse was recorded until 150 days after treatment of LW-AFC. The data showed the treatment of LW-AFC increased the average life span (Figure 1C) and median survival time (Figure 1D) in 12-month-old SAMR1 mice but melatonin not. While treatment with melatonin or LW-AFC failed to produce a significant increase in the average life span (Figure 1C) and median survival time (Figure 1D) in 24-month-old SAMR1 mice. The average weight of 12- and 24-month-old SAMR1 mice showed significantly increased after being treated with LW-AFC, while the treatment of melatonin decreased weight in 12-month-old SAMR1 mice and increased in 24-month-old SAMR1 mice (Figure 1E and 1F).

### The treatment of LW-AFC improved spatial memory defects of old SAMR1 mice

**Figure 2:**
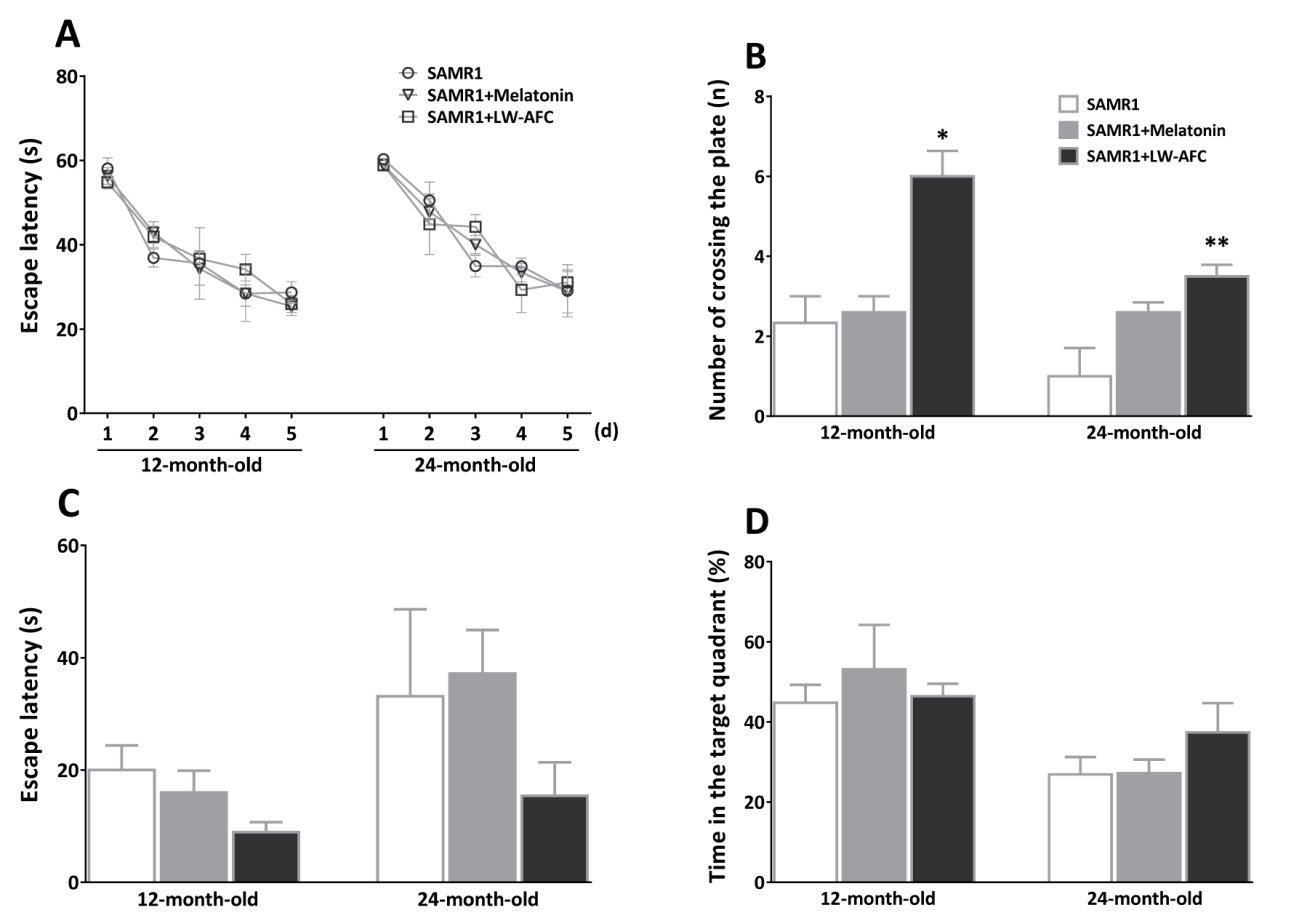
Effect of LW-AFC on the spatial learning and memory ability of SAMR1 mice The Morris water maze test was used. **A.** Escape latency, the first time that the mice crossed the former platform in the learning task. **B.** Numbers that the mice crossed the removed platform in the probe trial. **C.** Escape latency, the first time that the mice crossed the removed platform in the probe trial. **D.** The percent of swimming time within the target quadrant in the probe trial. The values are mean ± SEM. *n* = 3∼9. *P < 0.05, **P < 0.01, *versus* the SAMR1 mice by one-way ANOVA analysis followed by Dunnett's post hoc test.

To examine the ability of spatial learning and memory of SAMR1 mice, Morris water maze test was employed. In the learning task (Figure 2), there was no significant difference between the escape latency of each group. In the probe trial, the number of crossing the plate of 12- and 24-month-old SAMR1 mice was significantly increased by LW-AFC administration, escape latency and time in the target quadrant were not significantly different among groups. We did not find the effects of melatonin on spatial learning and memory defects in SAMR1 mice. This data indicated that LW-AFC administration significantly improved spatial memory of 12- and 24-month-old SAMR1 mice.

### Reversal of immunosenescence in old SAMR1 mice by LW-AFC

#### LW-AFC had enhancive effect on proliferation of splenocytes

**Figure 3:**
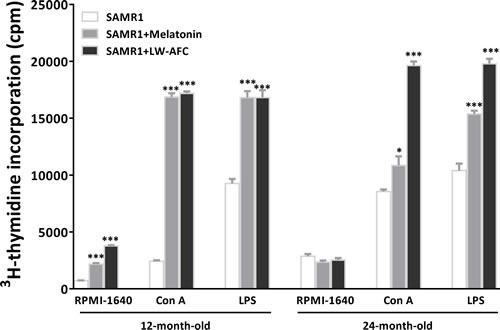
Effect of LW-AFC on the proliferation of splenocytes in SAMR1 mice 5 × 10^5^ spleen cells isolated from mouse (*n* = 2∼5) spleen were harvested, divided into three parts, and incubated with or without Con A / LPS for 56 h. Then 1μCi ^3^[H]-TdR was added each well, and cpm was read after another 16 h incubation. The values are mean ± SEM. *n* = 3. *P < 0.05, **P < 0.01, ***P < 0.001, *versus* the SAMR1 mice by one-way ANOVA analysis followed by Dunnett's post hoc test. Abbreviations used in the figure: ConA, concanavalin A; LPS, lipopolysaccharide.

To observe splenocyte proliferation of old SAMR1 mice, spontaneous, ConA- and LPS-induced splenocyte proliferation were investigated by ^3^H-thymidine incorporation. Results showed that the LW-AFC or melatonin treatment significantly increased the spontaneous splenocyte proliferation of 12-month-old SAMR1 mice (Figure 3). The treatment of LW-AFC or melatonin significantly increased ConA- and LPS-induced splenocyte proliferation in 12- and 24-month-old SAMR1 mice (Figure 3). This indicated that the function of T and B cell was enhanced by the treatment of LW-AFC.

#### LW-AFC had corrective effect on the disorder of lymphocyte subsets

**Figure 4:**
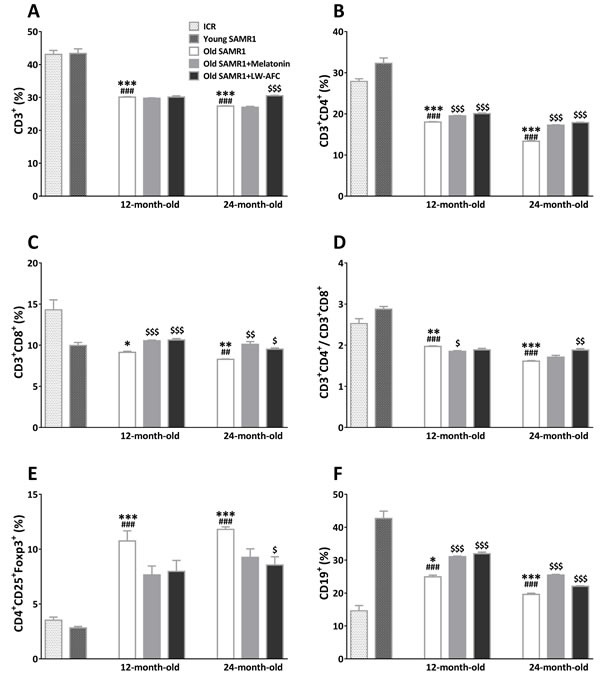
Effect of LW-AFC on the subsets of spleen lymphocytes in SAMR1 mice Flow cytometric analysis of **A.** CD3^+^ T cells, **B.** CD3^+^ CD4^+^ T cells, **C.** CD3^+^ CD8^+^ T cells, **D.** ratio of CD3^+^ CD4^+^ T cells and CD3^+^ CD8^+^ T cells **E.** CD4^+^ CD25^+^Foxp3^+^T cells, and **F.** CD19^+^ B cells in the spleen supernatant of mice. 5 × 10^5^ spleen cells isolated from mouse (*n* = 2∼6) spleen were harvested, divided into three parts, washed, and followed by incubation with antibodies, then quantified by flow cytometry, the sample size of the flow cytometric analysis was three. The values are mean ± SEM. *n* = 2∼16. *P < 0.05, **P < 0.01, ***P < 0.001, *versus* ICR mice; ^##^*P* < 0.01, ^###^*P* < 0.001, *versus* young SAMR1 mice; ^$^*P* < 0.05, ^$$^*P* < 0.01, ^$$$^*P* < 0.001, *versus* the old SAMR1 mice by unpaired Student's *t*-test and one-way ANOVA analysis followed by Dunnett's post hoc test.

In order to investigate the effect of LW-AFC on immunodeficiency, we detected the blood lymphocyte subsets in old SAMR1 mice. Regarding with T lymphocyte subsets, CD3^+^ T cells (Figure 4A), CD3^+^ CD4^+^ T cells (Figure 4B), ratio of CD4^+^ and CD8^+^ T cells (Figure 4D), in 12- and 24-month-old SAMR1 mice were significantly less than that in 7-month-old ICR and SAMR1 mice, and CD4^+^CD25^+^Foxp3^+^ T cells were significantly more than that in young mice (Figure 4E). The CD3^+^ CD8^+^ T cells of 12-month-old SAMR1 mice were only significantly decreased compared with ICR mice, and that of 24-month-old SAMR1 mice were significantly less than both young mice (Figure 4C). Treatment with melatonin was found to be able to reverse CD3^+^ CD4^+^ and CD3^+^ CD8^+^ T cells of 12- and 24-month-old SAMR1 mice, ratio of CD4^+^ and CD8^+^ T cells of 12-month-old SAMR1 mice, and CD3^+^ CD25^+^Foxp3^+^T cells of 24-month-old SAMR1 mice (Figure 4B, 4C, 4D and 4E). The CD3^+^ T cells, ratio of CD4^+^ and CD8^+^ T cells, CD3^+^ CD25^+^Foxp3^+^T cells of 24-month-old SAMR1 mice were improved by LW-AFC administration, CD3^+^ CD4^+^ and CD3^+^ CD8^+^ T cells of both old SAMR1 mice were also reversed by LW-AFC (Figure 4A, 4B, 4C, 4D and 4E). The B cells of 12- and 24-month-old SAMR1 mice were significantly more than that in ICR mice, and less than 7-month-old SAMR1 mice, while after treatment with LW-AFC and melatonin, B cells of both old SAMR1 mice were increased compared with control groups (Figure 4F). This indicated that the lymphocyte subsets in old SAMR1 mice were disorder and the treatment of LW-AFC and melatonin corrected aberrant lymphocyte subsets of old SAMR1 mice.

#### LW-AFC had regulatory effect on abnormal production of cytokines

**Figure 5:**
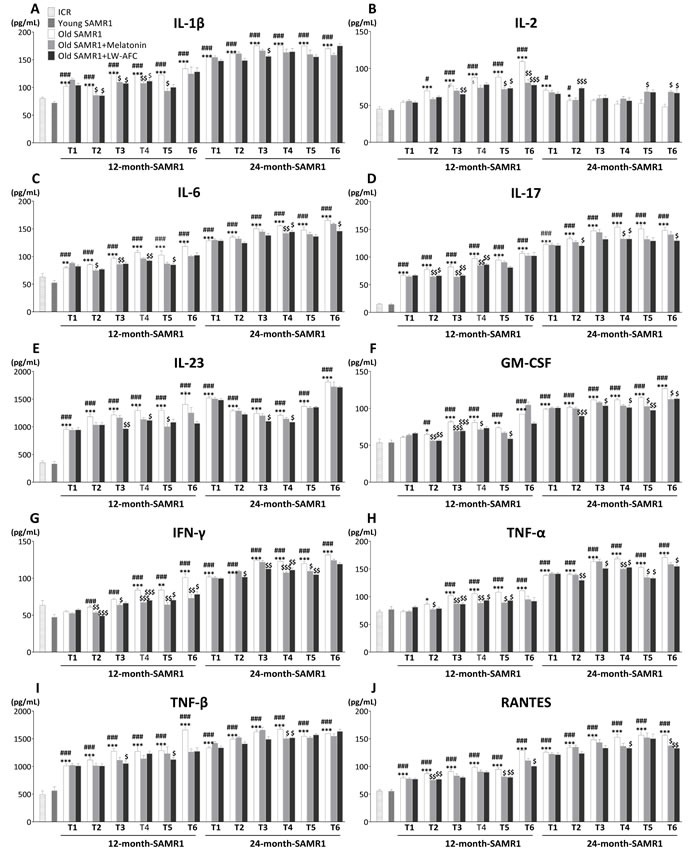

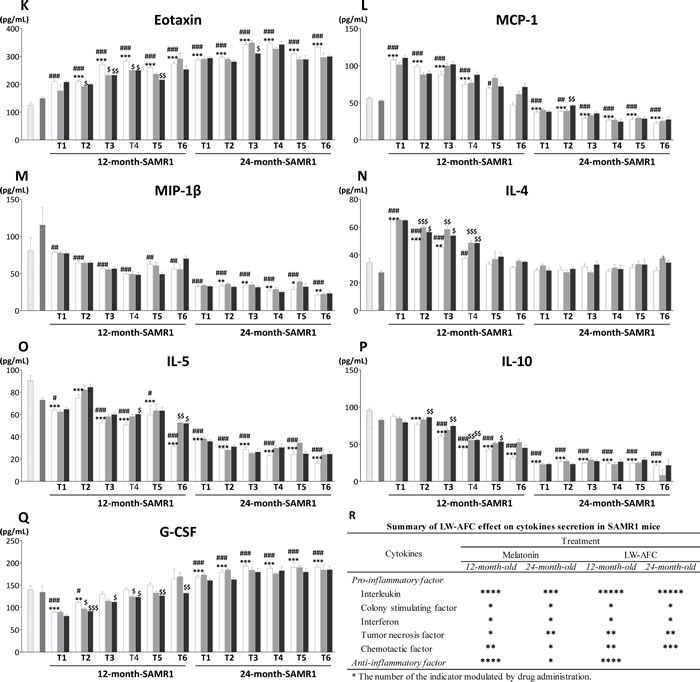
Effect of LW-AFC on the cytokines in the plasma of SAMR1 mice Concentrations (pg/mL) of **A.** interleukin-1β (IL-1β), **B.** interleukin-2 (IL-2), **C.** interleukin-6 (IL-6), **D.** interleukin-17 (IL-17), **E.** interleukin-23 (IL-23), **F.** granulocyte-macrophage colony stimulating factor (GM-CSF), **G.** interferon-γ (IFN-γ), **H.** tumor necrosis factor α (TNF-α), **I.** tumor necrosis factor β (TNF-β), **J.** regulated upon activation normal T cell expressed and secreted factor (RANTES), **K.** eotaxin, **L.** monocyte chemotactic protein-1 (MCP-1), **M.** macrophage inflammatory protein-1β (MIP-1β), **N.** interleukin-4 (IL-4), **O.** interleukin-5 (IL-5), **P.** interleukin-10 (IL-10) and **Q.** granulocyte colony stimulating factor (G-CSF) in the blood plasma were detected using Luminex^®^ X-MAP^®^ technology. T1∼T6 represent pre-administration, after 1∼5 months of administration respectively. **R.** Summary of LW-AFC effect on cytokines secretion in SAMR1 mice. The values are mean ± SEM. *n* = 2∼16. *P < 0.05, **P < 0.01, ***P < 0.001, *versus* ICR mice; ^#^*P* < 0.05, ^##^*P* < 0.01, ^###^*P* < 0.001, *versus* young SAMR1 mice; ^$^*P* < 0.05, ^$$^*P* < 0.01, ^$$$^*P* < 0.001, *versus* the old SAMR1 mice by unpaired Student's *t*-test and one-way ANOVA analysis followed by Dunnett's post hoc test.

In order to investigate the effect of LW-AFC on chronic inflammation, the levels of a number of cytokines (IL-1β, IL-2, IL-6, IL-17, IL-23, GM-CSF, IFN-γ, TNF-α, TNF-β, RANTES, eotaxin, MCP-1, MIP-1β, IL-4, IL-5, IL-10 and G-CSF) in blood were analyzed by multiplex bead analysis. The results (Figure 5) showed that some cytokines (IL-1β, IL-6, IL-17, GM-CSF, IFN-γ, TNF-α, TNF-β, RANTES, eotaxin) were significantly elevated, and some (MIP-1β, MCP-1, IL-23, IL-4, IL-5, IL-10) were significantly increased with ageing. In addition, the level of IL-2 in SAMR1 mice gradually increased since 13 months of age compared with 7-month-old ICR and SAMR1 mice, and it was no significant difference from 26 months of age (Figure 5B). Instead, G-CSF in SAMR1 mice was lower than 7-month-old ICR and SAMR1 mice at 12 and 13 months of age, and significantly increased from 24 months of age (Figure 5Q). After being administered with LW-AFC, 11 of 13 pro-inflammatory cytokines (eotaxin, GM-CSF, IFN-γ, IL-1β, IL-2, IL-6, IL-17, RANTES, TNF-α, IL-23, TNF-β) and all anti-inflammatory cytokines (G-CSF, IL-4, IL-5, IL-10) examined of 12-month-old SAMR1 mice were ameliorated, 12 of 13 pro-inflammatory cytokines (eotaxin, GM-CSF, IFN-γ, IL-1β, IL-2, IL-6, IL-17, MCP-1, RANTES, TNF-α, IL-23, TNF-β) and no anti-inflammatory cytokines of 24-month-old SAMR1 mice were modulated (Figure 5R). Melatonin was able to regulate 9 of 13 pro-inflammatory cytokines (eotaxin, GM-CSF, IFN-γ, IL-1β, IL-2, IL-6, IL-17, RANTES, TNF-α) and all anti-inflammatory cytokines (G-CSF, IL-4, IL-5, IL-10) examined of 12-month-old SAMR1 mice, and 8 of 13 pro-inflammatory cytokines (GM-CSF, IFN-γ, IL-2, IL-6, IL-17, RANTES, TNF-α, TNF-β) and 1 of 4 anti-inflammatory cytokines (IL-4) of 24-month-old SAMR1 mice (Figure 5R). This data indicated the treatment of LW-AFC could modulate the disordered secretion of cytokines in old SAMR1 mice.

#### LW-AFC had improvement effect on the phenotype of chronic inflammation

**Figure 6:**
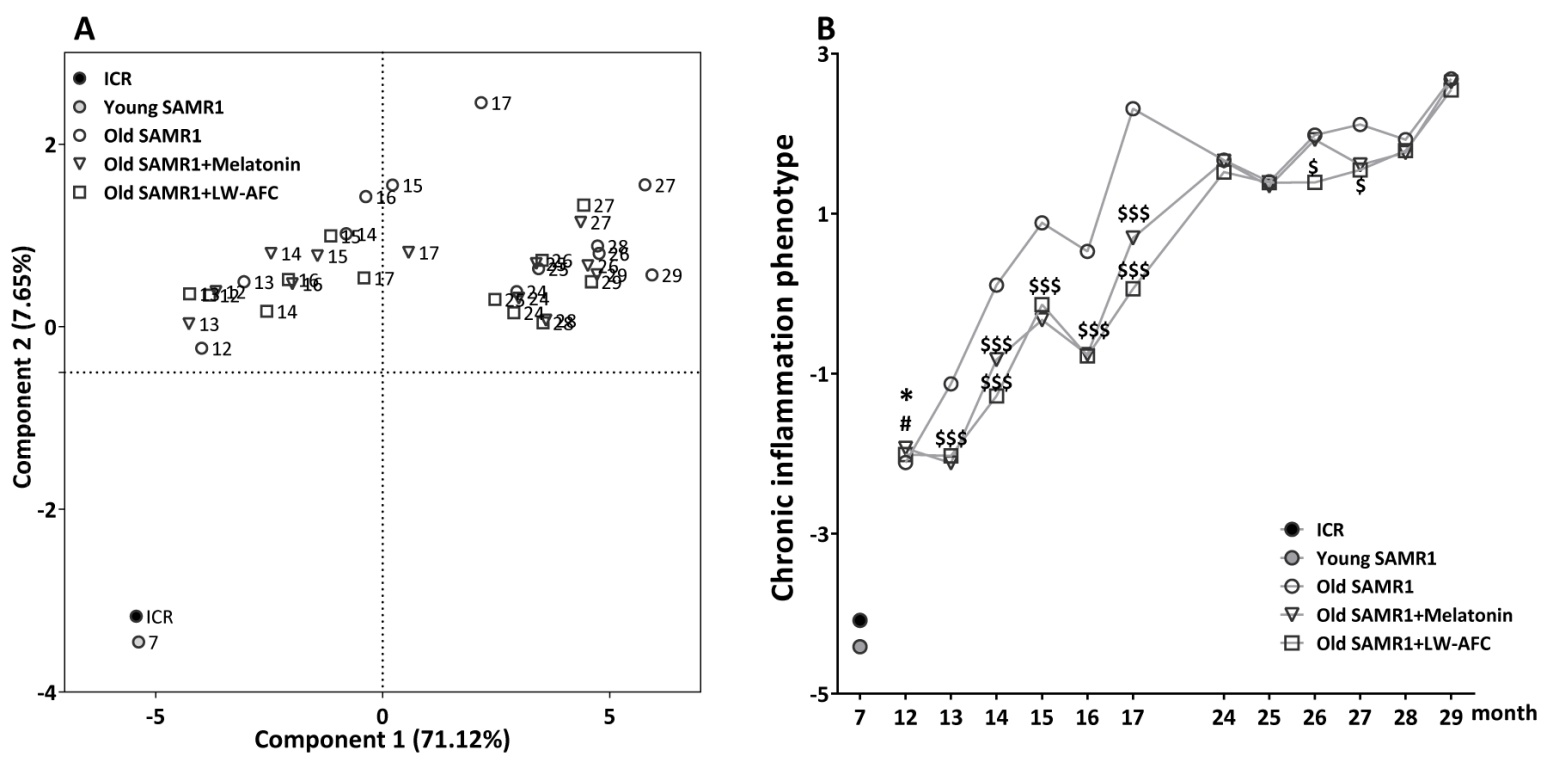
Principal component analysis of SAMR1 mice based on the phenotype of chronic inflammation **A.** PCA based on cytokine of SAMR1 mice. Each axis was derived by principal component analysis (x: Component 1 and y: Component 2). Each point represents one of SAMR1 mice, the number of each point represents month-age of mice. Component 1(variance explained: 71.12%), component 2 (variance explained: 7.65%) considered significant variance with a load below or equal to 0.50 (absolute value). Data represent only mean, *n* = 428, principal component analysis by SAS 9.2 statistics package, the significance level was set at *P* < 0.05. **B.** The average scores of cytokine of SAMR1 in the principal component analysis. The values are mean. *n* = 2∼16. *P < 0.05, *versus* ICR mice; ^#^*P* < 0.05, *versus* young SAMR1 mice; ^$^*P* < 0.05, ^$$^*P* < 0.01, ^$$$^*P* < 0.001, *versus* the old SAMR1 mice; Unpaired student's *t*-test and one-way ANOVA analysis followed by Dunnett's post hoc test.

In order to distinguish the immune phenotype of SAMR1 of varied age after treatment, principal component analysis was performed in this study. Principal component analysis (PCA) based on cytokine of ICR and SAMR1 mice showed that principal components 1 (PC1) and principal components 2 (PC2) classified mice of different age into three different regions (Figure 6A). Seven-month-old ICR and SAMR1 mice at bottom-left quadrant, most of 12∼17 month-old SAMR1 mice at top-left quadrant, and 24∼29 month-old SAMR1 mice were at top-right quadrant. The space distances of administered 13∼17 month-old SAMR1 location coordinates were nearer to ICR mice and young SAMR1 mice compared with their control SAMR1 mice of the same age. Nevertheless, the improvement effect on immune phenotype of 24∼29 month-old SAMR1 mice by melatonin and LW-AFC were less obvious than that of 13∼17 month-old SAMR1. For further analyzed anti-aging effect of LW-AFC, the average cytokine scores of SAMR1 mice in PCA were graphically displayed in Figure 6B. The plot showed that the average cytokine score of 12-month-old SAMR1 mice begin to significantly decrease after treatment with melatonin or LW-AFC for 1 month. The average cytokine score significantly decreased after treatment for 2 months in 24-month-old SAMR1 mice, while the anti-aging effect of melatonin or LW-AFC was not obvious after administration for 4 months. This indicated that LW-AFC had anti-chronic inflammatory activity and anti-inflammatory effects of LW-AFC in early-stage (12 months of age) were more effective than that in the advanced stage (24 months of age).

## DISCUSSION

One of the major features of human immunosenescence is immunodeficiency, because of thymic involution characterized by a progressive, age-related thymic epithelial cell loss and thymopoiesis impairment of the thymus [50–53]. The effectiveness of the immune response descends with age, specifically in the latter stages of life [54–56]. Among the multiple complex factors that contribute to the age-associated changes of immunological function in humans, the decline in CD4^+^ and CD8^+^ T-cell responsiveness is one of the most profound and consistent [57, 58]. A hallmark of age-related hypoimmunity is reduced humoral responses, and CD4^+^ T cells play a central role in the humoral response through their B cell helper functions [58]. Similar to CD4^+^ T cells, with increasing age there is a deterioration in CD8^+^ T-cell responsiveness to newly encountered antigens in older individuals [59]. CD4^+^CD25^+^Foxp3^+^ regulatory T cells (Treg), play important roles in both the immune system and the nervous system. The accumulation Tregs with advanced age, which in turn could deteriorate cytotoxic activity of CD8^+^ T and NK cells [60], was reported as a neuroprotective immunomodulator in AD [61–63]. The humoral immune response maintained by the B cell compartment are waned with age [64]. The percentage and numbers of total B cells, as defined by CD19 expression, decrease with age [65–67]. Aging also leads to the limited diversity of B cells that are available to respond to infection or challenge [65]. Therefore, deficiency of these lymphocytes will pose the aged organisms with the risk of new pathogen invasion and raised susceptibility to infection. In the present experiment, the administration of LW-AFC significantly increased the deduction of CD4^+^, CD8^+^ T-cell, CD4/CD8 ratio and CD19^+^ B-cell in the peripheral blood in old SAMR1 mice. Besides the lymphocyte quantities, the body resistance against infection, inflammatory illnesses or senescence also rely on the proliferation activity of lymphocytes. Studies have shown that obstruction of T lymphocyte proliferation may contribute to immunodeficiency related increasing age [68]. Our data also demonstrated that LW-AFC could augment the splenocyte proliferation in old SAMR1 mice. Based on modulating lymphocyte subsets of helper T cells, suppressor T cells, B cells, and regulatory T cells, and enhancing splenocyte proliferation, we inferred that restoring immunodeficiency may be one way for anti-aging of LW-AFC.

Accumulating studies show that senescence is not simply manifested as immunodeficiency, but a profound modification within cytokines as well [69–71]. The typical feature of this modification is a general raise in plasmatic levels and cell ability to secrete pro-inflammatory cytokines, leading to a chronic, low-grade, pro-inflammatory condition named “inflammaging” [27, 29, 72, 73]. Elevated concentration of pro-inflammatory cytokines and decreased level anti-inflammatory cytokines are correlated with an increased risk of morbidity and mortality in elderly. For example, elevated levels of IL-6 are associated with impaired functioning in the activities of daily living [74], and slower gait velocity [75]. IL-6 also stimulates osteoclastogenesis and osteoclastic activity, while shortening osteoblast survival [76–81]. With aging, inflammatory cells release pro-inflammatory cytokines lead to overexpression of adhesion molecules and procoagulant agents [82]. Local arterial vasoconstriction could be induced by TNF-α, IL-6 and IL-10 through the impact on the endothelium and synthesis of nitric acid [83]. In addition, IL-1β, IL-2, IL-6, and TNF-α modify endothelial function, which leading to prothrombotic state with inhibition of fibrinolysis and activation of coagulation pathways [84]. Furthermore, IL-2, IL-17, IL-23, TNF-β, GM-CSF, MCP-1, RANTES, eotaxin, IL-4, IL-5, IL-10 and G-CSF are also closely related with cognitive decline with ageing, synaptic or neuronal loss [85–91], osteoarthritis [92, 93] cancer [94], lung senescence [95]. Moreover, disturbance of cytokines are involved with nutritional status [96–98], bone metabolism [76, 79–81, 99] and muscle metabolism [100–102] of old individuals. In the present study, LW-AFC could regulated the abnormal level of pro-inflammatory cytokines IL-1β, IL-2, IL-6, IL-17, IL-23, GM-CSF, IFN-γ, TNF-α, TNF-β, RANTES, eotaxin, MCP-1, and anti-inflammatory cytokines IL-4, IL-5, IL-10 and G-CSF in old SAMR1 mice, and this suggested that anti-aging effects of LW-AFC may be *via* decreasing chronic inflammation.

In previous study, the grading scores increased steadily with advancing age in SAMR1 mice, the grading scores in SAMR1 mice levelled out at 24 months of age (∼8.2) were significantly higher than that at 12 months of age (∼3.0) [10]. And our results were consistent with previous reports (10.69 ± 2.53 *vs.* 7.25 ± 2.24, *P* < 0.0001). Moreover, the quantity of CD3^+^ CD4^+^ T cells (*P* < 0.0001), CD3^+^ CD8^+^ T cells (*P* = 0.0026), ratio of CD4^+^ and CD8^+^ T cells (*P* < 0.0001), and CD19^+^ B cells cells (*P* = 0.0011) of 12-month-old SAMR1 mice were significantly higher than 24-month-old SAMR1. These indicated that immunodeficiency degree of 12-month-old SAMR1 mice was higher than 24-month-old SAMR1. Furthermore, the chronic inflammation phenotype between the two time points of SAMR1 mice. The degree of chronic inflammation in 12-month-old SAMR1 mice (−2.11 ± 1.94) was lower than 24-month-old SAMR1 mice (1.67 ± 1.38). Therefore, 24-month-old SAMR1 mice were not only older than 12-month-old SAMR1 mice in physiological age, but also in immunodeficiency and chronic inflammation. The high-grade immunodeficiency and chronic inflammation in 24-month-old SAMR1 mice caused irreversible physiological aging. This might be the reason why anti-aging effectiveness of LW-AFC on 24-month-old SAMR1 mice was inferior to 12 months of age.

Taken together, our data demonstrated that LW-AFC possessed anti-aging activity and this effects might be through reversing immunodeficiency and decreasing chronic inflammation. LW-AFC may be a potential therapeutic agent for aging.

## MATERIALS AND METHODS

### Preparation of LW-AFC and HPLC analysis

LW-AFC was prepared from Liuwei Dihuang decoction and includes polysaccharide fraction (LWB-B), glycosides fraction (LWD-b) and oligosaccharide fraction (CA-30). Liuwei Dihuang decoction was prepared as previously described in Yang's [103], Zhang's [104, 105] and Cheng's [106, 107] study. After the prepared Liuwei Dihuang decoction was filtered by 6-layer gauze, the extraction solutions was centrifuged. The supernatant was concentrated into quintessence. The quintessence was extracted in ethanol to produce the supernatant (LWD), the sedimentation left in deionized water was concentrated into dried polysaccharide fraction (LWB-B). The ethanol elution fraction of the LWD, dissolved by macroporous adsorptive resins, to obtain glycosides component (LWD-b). And the water elution fraction of the LWD, dissolved by active carbon absorption column, to obtain oligosaccharide component (CA-30). LW-AFC was composed of 20.3% polysaccharide component (LWB-B), 15.1% glycosides component (LWD-b) and 64.6% oligosaccharide component (CA-30) in the dry weight ratio.

The components of LW-AFC were analyzed using HPLC. Briefly, for mixture of CA-30 and LWD-b, the chromatographic separation was obtained on a Diamond C18 column, there are 17 chromatogram peaks in fingerprint of CA-30 and LWD-b mixture. For LWB-B, the chromatographic separation was obtained on a NucleosilNH_2_ 100Å column, there were five chromatogram peaks and these five peaks represent fructose, glucose, sucrose, mannotriose and stachyose, the retention times of them were 6.260 min, 6.829 min, 8.186 min, 18.305 min and 21.506 min, respectively.

### Animals and drug administration

Original SAMR1 were kindly provided by Dr. T. Takeda at Kyoto University, Japan. And ICR-CD1 mice, a healthy and clean strain commonly used as control mice. The mice were kept in plastic-bottomed cages (290×180×152 mm) and maintained in the Beijing Institute of Pharmacology and Toxicology under standard housing conditions (room temperature 22±1°C and humidity of 55±5%) with a 12-h light/12-h dark cycle, and were fed pellet food (provided by the animal center of the AMMS). They were allowed free access to water and food. Two or three mice were kept in each cage. Each mouse was identified by coloring its fur with picric acid. All behavioral tests were performed between 19:00 p.m. and 6:00 a.m. (Beijing time). The animal treatment, husbandry and experimental protocols in this study were approved by the Institute of Animal Care and Use Committee (IACUC) of the National Beijing Center for Drug Safety Evaluation and Research (NBCDSER).

Twelve and twenty-four months SAMR1 mice were separated into 6 groups at random: 4 treatment groups, 2 SAMR1 control group, and each group had 16 mice (half male and half female). Seven-month-old SAMR1 and ICR-CD1 mice were served as young control (10 males). LW-AFC was dissolved in distilled water at 160 mg/mL, melatonin (Sigma Chemical Co. St. Louis, MO, USA) to 0.1 mg/mL. The mice in drug treated groups were given intragastric administration of melatonin or LW-AFC (0.1 mL/10 g body weight) daily for 150 days. SAMR1 mice as control group and the young control groups were given an equal volume of deionized water. The mice were weighed every 3 days, and evaluated the degree of senescence and locomotor activity every 15 days for 75 days. During the last two weeks of administration, all groups of animals were subjected to Morris water maze test. Following the behavioral experiment, the plasma was collected for cytokine, spleens for lymphocyte proliferation assay and lymphocytes subsets analysis.

### Grading score for evaluation of the degree of senescence

For evaluation of the degree of senescence in SAMR1 mice, a grading score system developed by Hosokawa, M., et al. (1984) [39] was adopted. The indexes in this system contained 11 categories selected from the clinical signs and gross lesions considered to be closely associated with the senescence process. In briefly, the grading score system, including reactivity, passivity, glossiness, coarseness, hair loss, ulcer, periophthalmic lesions, cataract, corneal ulcer, corneal opacity and lordoscoliosis, was designed to assess changes in behavior and appearance of the mice. The grade 0 represented no particular changes and grade 4 represented the most severe changes. Details are given in Supplement Table 1.

### Locomotor activity test

The procedure of locomotor activity test was according with Cheng, et al. (2011) [108]. Motor tracking was performed using a video-based behavior monitoring system (Jiliang Software Technology Co. Ltd., Shanghai, China). Each mouse was placed in an aluminum-plastic panel locomotor activity chamber (40cm×40cm×40cm, one animal pretesting cage). The total traveled distance of each mouse was recorded to indicate its spontaneous motor activity. The locomotor activity of each mouse was recorded for 20 min.

### Morris water maze test

The procedure of Morris water maze test was according to Charles V Vorhees & Michael T Williams (2006) [109]. The apparatus of Morris water maze test consisted of a 90cm in diameter and 45 cm circular pool with a black inner surfer, placed in a dim light sound-proof room. Unique geometric figures were installed on the curtains. The pool was divided into 4 quadrants and filled with 30 cm in depth 20±1°C water, a 6 cm in diameter black platform in the first quadrant. The platform was 1 cm below the surface of the water. This behavioral task included hidden-platform training (spatial learning) and probe trial (spatial memory) session. In the hidden-platform training session, the mouse was allowed 4 daily trials in the presence of the platform, for 5 subsequent days. In this session, mice were devoted into the pool facing the wall in one of the four quadrants. When the mouse located the platform, it was allowed to stay on the platform for 10 s, and if the mouse did not locate within 60 s, it was placed on the platform for 10 s to familiarize. After each trail, the mouse was taken back to its home cage and dried by warm towel. The experimental group sequence was changed every day for experimental data balance. In the probe trial session, the platform was removed, and the mouse was allowed to swim to search it for 60 s. During the whole Morris water maze test, the escape latency (the time taken to find the hidden platform) in hidden-platform training session, the escape latency (the first time that the mice crossed the removed platform), number of times that the mice crossed the removed platform and the swimming time within the target quadrant in probe trial session were recorded and analyzed.

### Flow cytometric analysis

Mouse spleen cells were harvested and divided into three parts. The first part of the spleen cells were treated with 100μL of 20μg/mL FITC anti-mouse CD3 antibody (BioLegend, San Diego, USA), 100μL of12.5μg/mL Percp anti-mouse CD4 antibody (BioLegend) and 100μL of 12.5μg/mL APC anti-mouse CD8 antibody (BioLegend) at 25°C for 30 min, washed, and followed by incubation with 100μL of 10μg/mL FITC-conjugated goat anti-rat IgG (BioLegend) and incubated at 25°C for 30 min in the dark. The second part of the spleen cells were treated with 100μL of 20μg/mL FITC anti-mouse CD3 antibody (BioLegend), 100μL of 12.5μg/mL Percp anti-mouse CD4 antibody (BioLegend),100μL of 25μg/mL APC anti-mouse CD25 antibody (BioLegend) and 100μL of 20 μg/mL PE anti- mouse FOXP3 (BioLegend) using the same protocol as above. The third part of spleen cells were treated with 100μL of 20μg/mL FITC anti-mouse CD19 antibody (BioLegend) and 100μL of 50μg/mL PE anti-mouse CD80 antibody (BioLegend) using the same protocol as above. After incubation, the cells were washed and resuspended in 0.5 mL of PBS/2% paraformaldehyde, then quantified by flow cytometry (BD Calibur™).

### Spleen cells and T, B lymphocytes proliferation assay

The mouse spleen was minced through a 40μm nylon cell strainer to acquire single cell suspension. Red blood cells were depleted by Tris-NH_4_Cl lysis buffer (0.017 M Tris-HCl, 0.144 M NH_4_Cl). According to the manufacturer's protocol, splenocytes were seeded in 96-wells plate at 5 × 10^5^ cells per well and stimulated with or without 0.5μg/mL concanavalin A (Con A) (Sigma-Aldrich, St. Louis, MO) or5μg/mL lipopolysaccharide (LPS) (Sigma-Aldrich), respectively. The lymphocytes were cultured in RPMI-1640 (GibcoBRL, Life Technologies, USA) medium, and replenished with 10% FBS (Hyclone Laboratories), streptomycin (100μg/mL) and penicillin (100 U/mL) at 37°C in a 5% CO_2_ humidified incubator for 56 h, and supplemented with 1μCi/well^3^H-thymidine (GEHealthcare, Buckinghamshire, UK) for the last 16 h. The cells were harvested on glass fiber filters by a Filtermate cell harvester (Packard). The total amount of ^3^H-thymidine incorporated in cells was measured by a β-scintillation counter (BECKMAN LS6500). The results were expressed as cpm (counters per minute) of stimulated and unstimulated cells.

### Multiplex bead analysis

Plasma samples of the mice were diluted 1:1 in the assay buffer and analyzed by multiplex bead analysis. Interleukin-1β (IL-1β), interleukin-2 (IL-2), interleukin-5 (IL-5), interleukin-17 (IL-17), interleukin-6 (IL-6), interleukin-4 (IL-4), interleukin-10 (IL-10), granulocyte-macrophage colony stimulating factor (GM-CSF), granulocyte colony stimulating factor (G-CSF), interferon-γ (INF-γ), tumor necrosis factor α (TNF-α), monocyte chemotactic protein-1 (MCP-1), regulated upon activation normal T cell expressed and secreted factor (RANTES), eotaxin, macrophage Inflammatory Protein-1β (MIP-1β), interleukin-23 (IL-23) and tumor necrosis factor β (TNF-β) were measured according to the manufacturer's instructions (Millipore Corp., USA). Briefly, the plasma samples were incubated with premixed beads overnight at 4°C, washed beads were further incurred with detection antibody for 1 h at room temperature followed by incubation with streptavidin-phycoerythrin for 30 min at room temperature. The samples were analyzed by Luminex 200™ (Luminex, Austin, TX, USA). The levels of IL-1β, IL-2, IL-5, IL-17, IL-6, IL-4, IL-10, GM-CSF, G-CSF, INF-γ, TNF-α, MCP-1, RANTES, Eotaxin, MIP-1β were detected by a multiplex map kit (MCYTOMAG-70K, Millipore),and IL-23, TNF-β were detected by another multiplex map kit (MGAMMAG-300K, Millipore).

### Principal component analysis

PCA is a classical multivariate technique, the aim is to extract the important information from numerous (n) possibly correlated variables (*M_1_, M_2_, …, M_n_*) and to represent it as a set of fewer variables, named principal components. The first PC (PC1) accounts for as much of the variability in the data as possible, and each succeeding component (PC2,…, PCk) accounts for as much of the remaining variability as possible [110]. PC are calculated from the eigen decomposition of covariance matrix M. The *j*th PC is a linear combination of the observed variables was calculated as follows:

$${\text{PC}}_{j}=\alpha_{1j}M_{1}+\ldots +\alpha_{nj}M_{n}$$

Where coefficients α*_nj_* are the elements of the eigenvector corresponding to *j*th eigenvalue.

In the present study, a data matrix with *m* observations on *k_1_, k_2_*and *k_3_* variables (*m* = 46, the number of individuals in the entire data set, *k_1_* = 4, cognitive markers, *k_2_* = 7, neuroendocrine markers, *k_3_* = 16, immune markers). PCA was carried out on M, scores of the selected PC were calculated for all individuals. We chose the PC1 and PC2 to plot, in order to distinguish young and old SAMR1 mice from chronic inflammation phenotype. The PCA was processed SAS software (SAS 9.2, SAS Institute Inc., Cary, NC), and visualized by GraphPad Prism^®^, version 6 (GraphPad software, San Diego, CA).

### Statistical analysis

All data are expressed as mean ± SEM. GraphPad Prism 6.0 (GraphPad Software, Inc., La Jolla, CA, USA) was used to plot and analyze data. Data between two groups were compared by Student's *t*-test. Comparisons of data from multiple groups against one group was analyzed by one-way analysis of variance (ANOVA) followed by Dunnett's post hoc test or two-way repeated-measures analysis of variance with Tukey multiple comparisons test. *P* < 0.05 was taken as statistically significant.

## SUPPLEMENTARY MATERIAL TABLE


