# Supplementary material for: The anti-aging effects of LW-AFC via correcting immune dysfunctions in senescence accelerated mouse resistant 1 (SAMR1) strain
Source: Oncotarget. 2016 Apr 20;7(19):26949–65. doi: 10.18632/oncotarget.8877 (PMC5053624; doi:10.18632/oncotarget.8877)
Supplement: Supplementary file 1 [file oncotarget-07-26949-s001.pdf]

The anti-aging effects of LW-AFC *via* correcting immune dysfunctions in senescence accelerated mouse resistant 1 (SAMR1) strain

Supplementary Material

Supplement Table 1 Criteria for grading score of senescence in mice

I) Behavior<br>1. Reactivity	The most intensive exploratory response observed within 30 seconds.	Grade 0<br>Natural behavior.	Grade 1<br>Abnormal grait with no lessening of agility and behavior patterns. Restlessness.	Grade 2<br>Definite decrease in agility and behavior patterns.	Grade 3<br>Does not move voluntarily but will move if nudge.	Grade 5<br>Immobile.
2. Passivity	Escape reaction from pinching of the nuchal skin or from hanging by the forelimb.	Natural escape reaction to pinching.	Decrease in escape reaction to pinching.	Loss of escape reaction to pinching. Preserved righting reaction to manual turn over.	Neither escape reaction to pinching nor righting reaction. Escape reaction to hanging by the forelimb.	Escape reaction nil
II) Appearance<br>(1) Skin and hair<br>1. Glossiness	Glossiness	Natural gloss.	Decrease in gloss.	Complete disappearance of gloss.	Complete disappearance of gloss and hair appears dirty.	Complete disappearance of gloss and hair very dirty.
2. Coarseness	Coarseness of hair on the head, nucha and dorsum determined according to the number of palpable, fine clumps of hair.	No coarseness,	Coarseness of less than an area of the head.	Coarseness of less than double the area of the head.	Coarseness of less than 3 times area of the head.	Coarseness of over 3 times area of the head.
3. Loss of ha	Loss or thinning of hair on the head, nucha and dorsum except for changes due to ulcer or periophthalmic lesion.	Neither loss or thinning of hair.	Loss of hair in less than an area of the head. Thinning of hair in less than 1/2 of total area.	Loss of hair in over one area of the head, less than in 1/4 of total area. Thinning of hair in more than 1/2 of total area.	Loss of hair in more than 1/4, in less than 1/2 of total area.	Loss of hair in over 1/2 of total area.
4. Skin ulcer	Ulcer or healed ulcer	No evidence of ulcer.	Healed ulcer or ulcer	Ulcer without healing	Ulcer without healing	Ulcer without healing

	on entire skin except for changed association with periophthalmic lesion.		with scab.	tendency, in less than one area of the head.	tendency in more one area of the head, in less than 1/4 area of all the skin.	tendency in more than 1/4 area of whole skin.
(2) Eyes<br>1. Periophthalmic lesion	Catarrhal changes in the periophthalmic area or swelling of the palpebral.	No changes.	Catarrhal changes limited to periophthalmic area or swelling of palpebral.	Catarrhal changes extending to nose.	Catarrhal changes extending further.	
2. Corneal opacity	Opaque changes of cornea with smooth surface by direct ophthalmoscopy.	No opacity.	Opacity with visible iris.	Opacity with visible iris. Positive retinal reflex by transillumination.	Opacity of entire cornea.	
3. Ulcer of the cornea	Opaque changes of cornea with rough surface by direct ophthalmoscopy.	No ulcer.	Linear ulcer corresponding to palpebral fissure.	Extension of ulcer over most of the area.	Ulcer of entire cornea.	
4. Cataract	Opaque changes of crystalline lens without retinal reflex by trans illumination. Impossible to score because of coexistence of grade 3 corneal opacity or ulcer.	Natural reflection.	Diminished reflection.	No reflection.		
(3) Spine<br>1. Lordokyphosis of the spine	Examined by inspection and palpation.	Natural anteroposterior curvature.	Increased curvature disappears with digital pressure on the dorsum.	Increased curvature disappears with a combination of manual cephalocaudal traction and digital pressure on the dorsum.	Permanent curvature.	
